# Nociceptive Responses and Resting-State Functional Connectivity of the Human Midbrain Tegmentum

**DOI:** 10.21203/rs.3.rs-10240247/v1

**Published:** 2026-07-16

**Authors:** Amanda Cao, Kiely Morris, Hanne van der Heijden, Raquel van Gool, Shantel Rose Gregory, Joseph Gonzalez-Heydrich, Daniel Paech, Sabina Berretta, Jaymin Upadhyay

**Affiliations:** Boston Children's Hospital; McLean Hospital; Boston Children's Hospital; Boston Children's Hospital; McLean Hospital; Boston Children's Hospital; Mass General Brigham; McLean Hospital; Boston Children's Hospital

**Keywords:** Rostromedial Tegmental Nucleus, Pain, Functional Magnetic Resonance Imaging, Resting-State Functional Connectivity, Postmortem, Mesocorticolimbic, Nigrostriatal

## Abstract

The rostromedial tegmental nucleus (RMTg) has been shown to inhibit midbrain dopaminergic centers through its GABAergic projections, and to be critical for regulating responses to aversive and rewarding stimuli. This nucleus has primarily been evaluated in rodents and non-human primates, while recent postmortem analysis of human brainstem tissue localized GABergic cell clusters in the putative RMTg. The objectives of this preliminary investigation in healthy controls (HC) were to (i) define RMTg function during evoked thermal stimulation and the resting-state using 7T functional magnetic resonance imaging (fMRI), and (ii) explore the expression of putative RMTg markers, μ-opioid receptors (OPMR1), nociceptin receptors (NOPR), and FOXP1 as well as tyrosine hydroxylase (TH) in the midbrain tegmentum. Relative to 40°C stimuli, greater responses to 46°C (normally noxious) in the putative RMTg as well as raphe nuclei, locus coeruleus, substantia nigra, thalamus, putamen, and anterior cingulate were observed. Resting-state 7T fMRI showed robust RMTg functional connectivity with components of mesocorticolimbic and nigrostriatal circuitry, thalamus, hippocampus, and cerebellar subdivision. In postmortem analyses, neurons immunolabeled for OPMR1, NOPR, and FOXP1 were detected in an area adjacent to the decussation of the superior cerebellar peduncle. These immune-positive neurons in the area of the putative RMTg were adjacent to intense TH immunoreactivity. This investigation provides novel evidence of activation of a distinct area within the human midbrain tegmentum, putatively identified as the RMTg, particularly during nociceptive processing in addition to its resting-state connectivity with the broader central nervous system. Further exploration of the human RMTg is warranted.

## Introduction

The rostromedial tegmental nucleus (RMTg), a mesopontine structure, has been shown in rodents and non-human primates to be a key node within valence encoding neural circuitry, specifically, within mesocorticolimbic and nigrostriatal pathways (Bourdy and Barrot 2012; Hong et al. 2011; Jhou et al. 2009; Li et al. 2019; Barrot et al. 2012). Preclinical research implicates the RMTg in the regulation of addictive behaviors, nociception, goal-directed behaviors, sensorimotor processing, and sleep (Bourdy and Barrot 2012; Hong et al. 2011; Jhou et al. 2009; Taylor et al. 2019; Sanchez-Catalan et al. 2017; Proulx et al. 2018). These compelling findings point to the RMTg as a potentially critical player in multiple psychiatric and neurological conditions.

The rodent RMTg receives projections from the cingulate, medial prefrontal cortex, habenula, among other brain regions, while RMTg GABAergic neurons inhibit dopaminergic tone through their projections to the ventral tegmental area (VTA) and substantia nigra (SN) (Bourdy and Barrot 2012). Thus, RMTg can be conceptualized as a braking system critical for encoding and responding to rewarding and aversive states by modulating dopaminergic tone. Such regulatory mechanisms are largely under the control of μ-opioid receptors (MOR), which are enriched in the rodent RMTg neurons (Steidl et al. 2017; Taylor et al. 2019). MOR activation inhibits the RMTg, causing a release of mesocorticolimbic and nigrostriatal dopaminergic neurons from the RMTg. Nociceptin receptors (NOPR), also present in the RMTg, play a similar role in inhibiting the RMTg and thus increasing the activity of dopaminergic neurons (Wasserman et al. 2016). Additionally, the transcription factor FOXP1 is selectively expressed in the RMTg and is implicated in sensorimotor processing, cognition, and other domains (Smith et al. 2019; Lahti et al. 2016; Trelles et al. 2021). Given that FOXP1 expression across the brain is consistently restricted to projection neurons rather than interneurons, its expression in the RMTg is consistent with the established identity of these cells as long-range GABAergic projection neurons (Smith et al. 2019; Fong et al. 2018; Precious et al. 2016; Tamura et al. 2004).

Currently, the molecular and functional properties of the human RMTg have not been widely investigated, hampering our understanding of the potential role of this nucleus in humans. Recently, Filimontseva et al. localized the GABergic cell clusters in the human post-mortem brainstem tissue in a location analogous to the rodent RMTg (Filimontseva et al. 2026). In the current investigation, we expand upon these important post-mortem findings and characterize this area and its circuitry in human using a combination of functional magnetic resonance imaging (fMRI) studies performed at 7 Tesla in healthy human subjects. 7 Tesla MRI was chosen to maximize spatial resolution and signal sensitivity for functional interrogation of the small and anatomically complex RMTg (A et al. 2017; Platt et al. 2021) region. The objectives of this work were to define nociceptive responses in the human RMTg by evaluating and comparing blood oxygenation level-dependent (BOLD) responses during normally 40°C and 46°C thermal stimulation, (ii) define resting-state, RMTg functional connectivity, and (iii) further inform human RMTg localization. In exploratory post-mortem analysis of healthy human donor brainstem tissue, we evaluated the expression of MOR, NOPR, FOXP1 and tyrosine hydroxylase (TH; a dopaminergic marker) expression in the midbrain tegmentum. This preliminary study in humans further provides compelling support to define the role of this midbrain area, putatively identified here as the RMTg.

## Materials and Methods

### In Vivo Neuroimaging

#### Study Participants

This study enrolled male and female participants between 18 and 55 years of age. Healthy controls (HC) without psychiatric illness and without first-degree relatives with a psychotic disorder were evaluated. HCs were free of any history of pain syndromes or any ongoing acute pain conditions during the study. All participants were in good physical health. Individuals with MRI contraindications, significant medical or neurological illness, a Diagnostic and Statistical Manual of Mental Disorders, 5th Edition (DSM-5) substance use disorder, or who were pregnant or nursing were excluded. All study procedures were approved by the MGB Institutional Review Board (IRB) and the Boston Children’s Hospital IRB. All participants provided written informed consent.

#### Quantitative Sensory Testing (QST)

In line with prior studies, QST was implemented to assess sensitivity to heat and cold stimuli in the current HC population (Upadhyay et al. 2015; Upadhyay et al. 2012; Golden et al. 2023). A thermode (1.6×1.6 cm^2^ surface area) was placed on the dorsum of the subjects’ right hand at a baseline temperature of 32°C. Subject-specific warm and cool detection thresholds, heat and cold pain detection thresholds, and heat and cold pain tolerances were determined. The Brief Pain Inventory (BPI) was use to confirm absence of any ongoing pain in HCs during study participation (Cleeland and Ryan 1994)).

#### Neuroimaging Acquisition

High-resolution structural and task-based functional MRI sequences were acquired for all participants using a 7T Siemens MAGNETOM Terra MRI system equipped with a Nova Medical single-transmit/32-channel receive (1Tx/32Rx) head coil, with acquisition parameters optimized for brainstem and mesopontine regions.

#### Structural Imaging

Structural imaging included both T1- and T2-weighted sequences. The T1-weighted images were acquired using a magnetization-prepared rapid gradient echo (MPRAGE) sequence with the following parameters: repetition time (TR) = 2530 ms; inversion time (TI) = 1100 ms; echo times (TE1/TE2) = 1.8/3.7 ms; flip angle = 7°; isotropic voxel resolution = 0.75 mm^3^; and a GRAPPA acceleration factor of 2. T2-weighted images were acquired using a turbo spin echo sequence with TR = 5000 ms; TE = 53 ms; flip angle = 180°; voxel resolution = 0.75 mm^3^; and GRAPPA acceleration factor = 2.

#### Functional MRI

Functional MRI (fMRI) data were acquired using a gradient-echo EPI sequence with the following parameters: TR = 2500 ms; TE = 23 ms; flip angle = 75°; 35 sagittal slices centered at the midline of the brainstem; isotropic voxel resolution = 1.0 mm^3^; field of view (FOV) = 192 × 192 mm^2^; GRAPPA acceleration factor = 5; echo spacing = 0.81 ms; and bandwidth = 1532 Hz/Px. For each subject, manual voltage and B_0_ shimming adjustments were performed prior to acquisition to ensure optimal signal quality over the brainstem. Furthermore, an advanced Dual Polarity GRAPPA-EPI reconstruction technique was applied to minimize artifacts and improve time-series stability [38,39]. Using this fMRI protocol, we calculated a temporal signal-to-noise ratio of ~ 24 for BOLD fMRI data at the midbrain and pons. A single-volume, whole brain, EPI scan with matching TR and TE was acquired for image registration purposes. During fMRI, thermal stimuli were applied to the dorsum of the right hand using a block design consisting of five 30-second off/15-second on cycles. During the off condition, a baseline temperature of 32°C was maintained. In Run 1 (~ 4 minutes), the on condition involved thermal stimulation at 40°C. In Run 2 (~ 4 minutes), stimulation at 46°C was applied. fMRI data where 40°C was administered was captured prior to the 46°C fMRI acquisition to avoid or introduce desensitization for 40°C stimuli. Following each run, participants rated their pain experience on a numerical pain scale ranging from 0 (no pain) to 10 (worst imaginable pain).

#### Thermal Stimulation fMRI Analyses

Thermal stimulation (40°C and 46°C) fMRI data were preprocessed and analyzed using FSL 6.0.7.18 FMRIB Software Library as recently described (Cay et al. 2025; Golden et al. 2024). However, in the current single-subject fMRI analyses, a 1 mm full width at half maximum (FWHM) smoothing kernel was applied. Briefly, a general linear model (GLM) was implemented using FSL FEAT to evaluate brain responses to thermal stimulation during Run1 (40°C) and Run2 (46°C) conditions. Additionally, subject-specific, single-volume, whole-brain fMRI and MPRAGE datasets were used to during image registration of thermal stimulation fMRI datasets to the Montreal Neurological Institute (MNI) template brain at 1mm^3^ resolution. After QA/QC of single-subject thermal stimulation fMRI datasets, preliminary group analyses were performed using a fixed effects analysis. Statistical thresholds were set to z > 3.1. Cluster-size correction was not applied owing to the small volume of the RMTg and other brainstem nuclei. Localization of the human RMTg as well as the lateral cluster (LatC) on the MNI template brain was guided by anatomical descriptions recently provided (Filimontseva et al. 2026). Additional landmarks to guide RMTg localization included the decussation of the superior cerebral peduncle, SN, VTA and red nucleus. The SN pars compacta, SN pars reticulata, and VTA regions of interest were anatomically defined in the MNI template brain using predefined templates (https://neurovault.org/images/786456/). The Harvard-Oxford Subcortical and Cortical Structural Atlases as well as the Talairach Atlas embedded within FSL were used to define ROIs for the red nucleus, putamen, nucleus accumbens, pallidum, anterior cingulate cortex, and medial prefrontal cortex. Thalamic parcellations were derived from the Thalamus Optimized Multi-Atlas Segmentation) atlas in MNI152 space (Baldacci et al. 2015; Saranathan et al. 2021; Su et al. 2019). Additionally, 2 mm-radius spherical ROIs were generated for the left and right habenula, centered at MNI coordinates (− 3, − 25, 2) and (5, − 25, 2), respectively, based on prior work (Dai et al. 2021). Furthermore, clusters of activation within the brainstem were identified using a combination of atlases and histological studies (Lavezzi et al. 2019; Naidich and Duvernoy 2009; Schira et al. 2023). BOLD responses at the ROI level and between 40°C and 46°C conditions were further evaluated by calculating Cohen’s *d* effect sizes. Spearman’s rank correlations were computed to determine ROI-to-ROI relationships between 40°C and 46°C stimulations.

##### 7T-Resting-State fMRI

Resting-state fMRI data were obtained from the Human Connectome Project Young Adult 7T dataset (HCP-YA) (Van Essen et al. 2013). Briefly, resting-state fMRI data from the HCP-YA 7T dataset were acquired on a Siemens 7T Magnetom scanner using a gradient-echo EPI sequence with the following parameters: TR = 1,000 ms, TE = 22.2 ms, flip angle = 45°, 85 axial slices, isotropic voxel resolution = 1.0 mm^3^; GRAPPA acceleration factor = 2. Four runs of 900 volumes each (~ 16 minutes per run) were acquired per participant. 80 healthy adults with complete four-run 7T resting-state acquisitions across both phase encoding directions (posterior-anterior and posterior-anterior) were initially selected from the HCP-YA dataset. Data were preprocessed using the HCP minimal preprocessing pipeline (Glasser et al. 2013) and analyses were performed using CONN 22.v2407 (Whitfield-Gabrieli and Nieto-Castanon 2012) and SPM 12.7771 (Friston 2007). During preprocessing analysis in CONN, data from two out of 80 HCP-YA datasets were excluded due to uncorrectable structural segmentation errors. Anatomical data were segmented into grey matter, white matter, and CSF tissue classes using SPM unified segmentation and normalization algorithm (Ashburner 2007; Ashburner and Friston 2005) with the default IXI-549 tissue probability map template. Denoising was performed and included: white matter and CSF signal regression (aCompCor, 5 components each), 24 motion parameters (6 rigid-body parameters, derivatives, and squares), linear detrending, spatial smoothing at 1 mm FWHM, and bandpass filtering (0.008–0.09 Hz) (Nieto-Castanon 2020). Outlier volumes were identified via ART-based scrubbing (framewise displacement > 0.2 mm; global signal z-score > 3).

Seed-based connectivity maps (SBC) and ROI-to-ROI connectivity matrices (RRC) were estimated characterizing the patterns of functional connectivity with 199 ROIs. RMTg seeds (1 mm sphere centered around MNI coordinates ± 3, −21, −19, max z-statistic value for 40°C vs. 46°C contrast) as well as additional anatomical landmarks employed in the task-based fMRI analysis. Functional connectivity strength was represented by Fisher-transformed bivariate correlation coefficients from a weighted-GLM (Nieto-Castanon 2020), defined separately for each pair of seed and target areas, modelling the association between their BOLD signal timeseries. To compensate for possible transient magnetization effects at the beginning of each run, individual scans were weighted by a step function convolved with an SPM canonical hemodynamic response function and rectified (Smith et al. 2013).

For SBC, mean seed time series were correlated with all brain voxels across four sessions, for the left and right RMTg individually, and Fisher’s r-to-z transformation was applied. Group-level inference used one-sample t-tests against zero(Nieto-Castanon 2020). Statistical thresholding employed threshold-free cluster enhancement (TFCE) with 5,000 permutations at p-FWE < 0.05. Cluster anatomy was labeled using the FSL Harvard-Oxford and AAL cerebellar atlases. RRC between the RMTg ROI and all target ROIs was assessed. Pairwise connectivity was estimated simultaneously across all ROI pairs. Statistical inference used one-sample t-tests against zero, thresholded at p-FWE < 0.05 corrected across the full connectivity matrix (Nieto-Castanon 2020).

### Post-mortem Assessment of the Midbrain

#### Human Postmortem Brain Tissue

Postmortem human brains from 5 unaffected donors (average age 52.25 yo; 2 female and 3 males) were obtained through the Harvard Brain Tissue Resource Center, part of the NeuroBioBank of the National Institutes of Health (NIH). Individual brains were obtained on a postmortem interval (PMI) of less than 48h. From the fresh frozen hemisphere, the midbrain was sectioned axially and cryosectioned axially at 18 um. Samples were stored at − 80°C on Superfrost Plus slides until used for immunohistochemical experiments.

#### Immunohistochemistry

Slides were removed from the − 80°C freezer and dried at room temperature for ~ 10min until tissue was fully dried against the slide. Tissue was fixed at room temperature in 4% paraformaldehyde (PFA) for 15 minutes. Immediately after, slides were washed with 0.01M PBS − 0.2%Triton-X (PBST). Slides were quenched for 15 min in 0.3% hydrogen peroxide and PBST. Slides were blocked with 2% bovine serum albumin (BSA fraction V Fisher #BP1605) made up in PBST for 1hr. The slides incubated 3 nights with primary antibody (1:500) in PBST at 4°C (**Table S1**). After primary incubation, the sections were incubated in secondary biotinylated horse-anti-rabbit or rabbit-anti-sheep IgG secondary antibody (1:500, Vector Laboratories) in PBST for 1 h followed by Streptavidin Horseradish Peroxidase (1:5000, Invitrogen). The proteins were visualized with 3,3′-diaminobenzidine (Sigma-Aldrich Company Ltd., Gillingham Dorset, UK) for 20 min. Finally, the sections were dehydrated in ethanol, cleared in xylene and cover-slipped. Additionally, we stained negative controls in the absence of the primary antibodies; no specific staining was apparent.

#### Microscopy

Entire tissue section images were collected via scanning with transmitted light at 20x using an Evident VS-200 slide scanning microscope, courtesy of the Neuroimaging Facility at Harvard Medical School.

## Results

### Study Participants

Twenty-two HCs (12 males, 10 females, mean ± standard deviation age: 32.59 ± 9.79 years) completed the study protocol including 7T fMRI (Table 1). QST experiments revealed warm/cool detection thresholds, heat pain/cold pain detection thresholds, and heat pain/cold pain tolerances in line with prior HC populations and thus indicates normal pain sensitivity (Golden et al. 2024; Cay et al. 2025). Group average responses on the BPI indicated little to no pain.

### Thermal Pain Responses Measured with 7T fMRI

**Figure 1:**
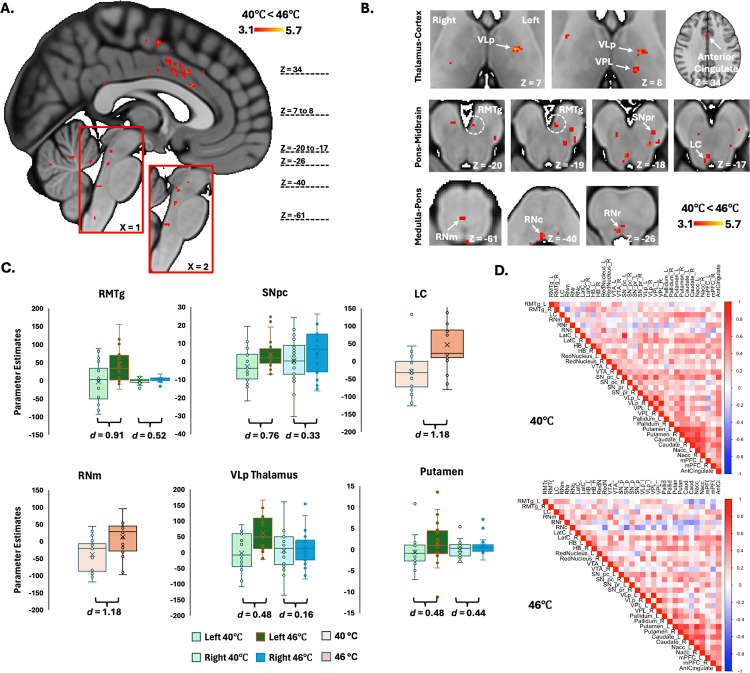
7T Thermal Stimulation (46°C vs. 40°C) fMRI in HCs. **(A)** Group level activation contrast for 46°C vs 40°C stimulation overlayed on an MNI template brain (z-stat threshold 3.1). **(B)** Clusters of higher activation for 46°C (mean pain rating of 6.02 ± 2.04 (0–10 pain scale)) vs. 40°C (mean pain rating of 2.93 ± 1.96 (0–10 pain scale)) stimulations in the cortex, thalamus, midbrain, pons, and medulla shown in the axial plane. **(C)** Parameter estimates extracted from a subset of clusters shown in panels **A**. and **B**. Cohen’s *d* effect sizes are reported. **(D)** Spearman correlation matrices of parameter estimates from ROIs within postulated RMTg circuitry during 40°C and 46°C stimulations. Positive correlations shown in red and negative correlations shown in blue. LatC (Lateral Cluster); mPFC (medial Prefrontal Cortex); NAcc (Nucleus Accumbens); LC (Locus Coeruleus); RNc (Raphe Nuclei caudal); RNm (Raphe Nuclei medulla; RNr (Raphe Nuclei rostral); RMTg (Rostromedial Tegmental Nucleus); SNpc (Substantia Nigra pars compacta); SNpr (Substantia Nigra pars reticulata); VLp Thalamus (Ventral Lateral Nucleus, Posterior division of the Thalamus); VPl (Ventral Posterolateral Nucleus of the Thalamus); VTA (Ventral Tegmental Area).

A comparison of BOLD responses between 40°C and 46°C revealed greater activity for 46°C in several brainstem, thalamic, striatal and cortical regions (Fig. 1A–C). Verbal pain ratings collected immediately after 40°C (fMRI Run 1) and 46°C (fMRI Run 2) stimulations confirmed that the latter was the more noxious of the two stimuli (Table 1). Two 46°C fMRI datasets were not included in the final analysis due to excessive motion artifact. Within the midline brainstem, multiple raphe nuclei clusters (i.e., medulla, caudal, and rostral segments) showed greater activity for the 46°C vs. 40°C. A similar pattern was present in mesopontine nuclei including the putative RMTg, locus coeruleus (LC), SN pars reticulata, ventral lateral nucleus, posterior division of the thalamus (VLp Thalamus), ventral posterolateral nucleus of the thalamus (VPl Thalamus), putamen, and anterior cingulate cortex. Interestingly, in parallel with the SN pars reticulata as well as VLp and VPl thalamic responses for 46°C vs. 40°C, greater RMTg responses for were quantified in the left hemisphere, contralateral to the thermal stimulation site (i.e., dorsum of the subjects’ right hand)), compared to the right. The within group average BOLD response maps for 46°C stimulation further demarcating the putative RMTg, LatC, SN, thalamus, among other regions are provided in **Figure S1**. Overall, BOLD responses for 46°C in the left (p = 0.95) or right VTA (p = 0.43) and other mesocorticolimbic structures were not identified, and thus points to activation being primarily elicited in nigrostriatal circuitry in HCs. In **Figure S2**, BOLD responses in the CNS using our 7T fMRI protocol for 46°C is shown for a single subject and demonstrates effective engagement of classical pain processing.

**Figure 2:**
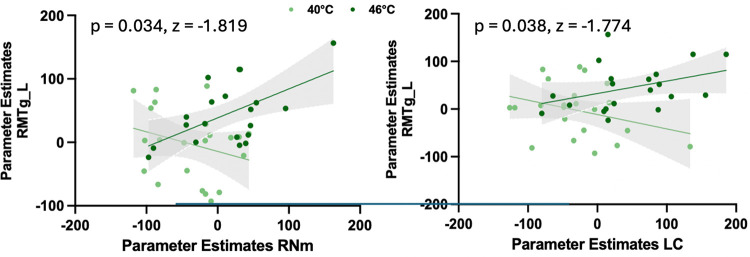
Associations between Brainstem Nuclei Activation with 7T Thermal Stimulation (46°C vs. 40°C) fMRI in HCs. Correlations between parameter estimates of Left RMTg (Rostromedial Tegmental Nucleus) and RNm (Raphe Nuclei medulla) and LC (Locus Coeruleus) for noxious (46°C; N=20) vs non-noxious (40°C; N=22) stimulation. RMTg – RNm at 40°C (Spearman’s r: −0.17); RMTg – RNm at 46°C: (r: 0.41). RMTg – LC at 40°C (r: −0.19); RMTg – LC at 46°C: (r: 0.38).

Variation of putative RMTg BOLD responses with other brainstem, thalamic, striatal, and cortical regions of interests (ROIs) and between 40°C vs. 46°C stimulations were explored (Fig. 1D). Of note, relative to the 40°C condition, an increase in correlation coefficients was present between the left RMTg and brainstem regions such as the raphe nuclei (medulla cluster) and LC during 46°C stimulation (Fig. 2). Correlations between the left RMTg and striatal regions (i.e., caudate and nucleus accumbens) showed a negative correlation for the 46°C condition but was not significantly different from 40°C. Significant changes in RMTg-VTA or RMTg-SNpc correlations in either hemisphere and between 40°C vs. 46°C stimulations were not observed (**Figure S3**).

### RMTg-based Resting-State Functional Connectivity using 7T fMRI

**Figure 3:**
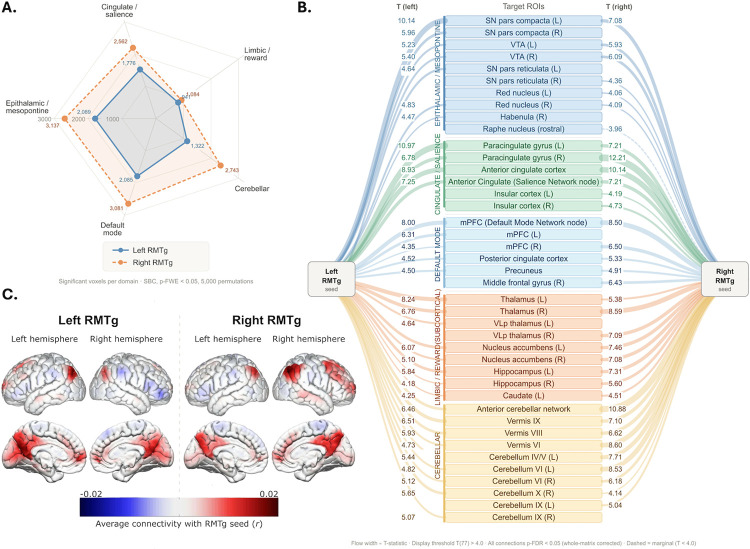
Resting-State Functional Connectivity of the Human RMTg. **(A)** Domain-level summary of SBC connectivity for the left (solid blue) and right (dashed orange) RMTg seeds. Spoke length represents the total number of significant voxels (p-FWE < 0.05) within each functional circuit domain. **(B)** ROI-to-ROI functional connectivity between the left and right RMTg seeds and target regions of interest, grouped by functional circuit. Flow width is proportional to the T-statistic. Only connections surviving p-FDR < 0.05 corrected across the full connectivity matrix are shown. Display threshold T(77) > 4.0; dashed flow indicates marginal connection (T < 4.0). **(C)** Cortical surface representation of RMTg seed-based correlation. Functional connectivity maps for the left and right RMTg seeds are displayed on inflated cortical surfaces, each shown as lateral (top) and medial (bottom) views of the left and right hemispheres. Color scale reflects average connectivity strength (r) with the RMTg seed, ranging from r = −0.02 (blue, negative connectivity) to r = 0.02 (red, positive connectivity). Maps are derived from seed-based correlation analysis with TFCE correction (p-FWE < 0.05, 5,000 permutations). FDR (false discovery rate); FWE (familywise error rate); mPFC (medial prefrontal cortex); RMTg (rostromedial tegmental nucleus); ROI = region of interest; SBC (seed-based correlation); SN (substantia nigra); TFCE (threshold-free cluster enhancement); VLp (ventral lateral posterior nucleus of the thalamus); VTA (ventral tegmental area). See also **Figure S4**.

Seed-based analyses revealed a consistent and anatomically coherent connectivity profile across left and right putative RMTg seeds (TFCE, p-FWE < 0.05, **Tables S2-S3**). Across both seeds, the RMTg showed robust positive functional connectivity with medial cortical regions, including the anterior and posterior cingulate cortex, paracingulate gyrus, medial prefrontal cortex, and precuneus, consistent with engagement of default mode and salience networks (Fig. 3). Subcortically, functional connectivity was observed with key components of mesolimbic and dopaminergic circuitry, including the SN, VTA, nucleus accumbens, thalamus, and hippocampus. Cerebellar connectivity was robust, spanning the vermis (lobules III–IX, most prominently lobules VIII–IX) and bilateral hemispheric regions, including lobules VI, V, VI, IX, and X. The right RMTg exhibited a modestly broader cortical profile, additionally engaging lateral parietal and frontal regions, including angular and supramarginal gyri, orbitofrontal cortex, insula, and inferior frontal gyrus. Negative functional connectivity was consistently observed with inferior temporal and language-related regions, including the supramarginal gyrus, inferior frontal gyrus, and posterior superior temporal gyrus (p-FDR < 0.05; T < 4.0). ROI-to-ROI analyses confirmed these patterns (p-FWE < 0.05), with strongest connectivity observed between the RMTg and dopaminergic midbrain targets, anterior/paracingulate cortex, and medial prefrontal default mode regions (**Table S4**). Corresponding functional connectivity maps on axial cross-sections and on the MNI template brain are provided in **Figure S4**.

### Postmortem Analysis

In the postmortem human brain samples, the neurons immunolabeled for OPMR1, NOPR, and FOXP1 were detected in a broad area adjacent to the decussation of the superior cerebellar peduncle. This area showed light cellular staining of OPRM1 and NOPR, and darker nuclear staining of FOXP1. Many cells also contained lipofuscin, a non-specific byproduct often detected in aging individuals (light brown staining in **Figure S4**). This location is consistent with previous reports from rodents and non-human primates, showing selective expression of these markers in the RMTg. As expected, immune-positive neurons were adjacent to areas of intense TH immunoreactivity. These results suggest that the location of the RMTg in the human brain may be similar to that shown in rodents and non-human primates. However, the distribution of neurons immunolabeled for FOXP1, OPRM1 and NOPR was highly variable across individuals and appears to be less circumscribed than previously described in other species, with sparse neurons intermingling with TH-positive areas. Overall, the sparsity of these neurons across the rostral tegmentum indicates that the human RMTg, based on the current molecular probes, may be integrated in a broader network of structures including the SN, VTA, among others.

## Discussion

The RMTg, a mesopontine inhibitory nucleus, has long been shown in non-human species to play a major role in the regulation of upstream dopaminergic signalling pathways during reward-aversion processing (Jhou et al. 2009; Bourdy and Barrot 2012; Taylor et al. 2019). To date, the RMTg has been primarily characterized in rodent and non-human primate species, yet very recent histological findings in human brainstem tissue have described the anatomical localization and cytoarchitectonic of GABAergic neurons in an area putatively corresponding to the RMTg (Filimontseva et al. 2026). Notably, GABAergic neurons are represented in several different midbrain tegmentum nuclei, making it a challenge to identify the RMTg uniquely on a molecular basis. The current study, through evoked thermal stimulation 7T fMRI methodology, translates prior preclinical and human postmortem findings to functionally delineate the putative RMTg in addition to other brainstem nuclei (i.e., raphe nuclei, LC, and SNpc), striatum, thalamic nuclei, and cortical hubs (anterior cingulate) implicated in the processing of thermal and nociceptive stimuli. Using resting state 7T fMRI data (Van Essen et al. 2013), the characterization of the RMTg was extended by describing its functional connectivity with midbrain targets (SN and VTA), the HB, and striatum as well as default-mode, salience and cerebellar networks.

In animal models, RMTg inhibition has been shown to yield pain hyposensitivity (Taylor et al. 2019), while RMTg activation, via habenula afferents, can underpin pain hypersensitivity (Liu et al. 2025). RMTg activation also reduces motivation to exert effort for reward (Proulx et al. 2018). The current neuroimaging findings indicate that during nociceptive processing (confirmed by subject-reported pain ratings particularly for 46°C stimuli), the human RMTg is also activated. In a murine model of neuropathic pain, the HB-RMTg-VTA pathway was implicated in the generation of an aberrant nociceptive state alongside affective and cognitive symptoms (Liu et al. 2025). In our HC fMRI studies, the VTA or nucleus accumbens was not activated at a significant level during an evoked pain state, yet BOLD responses within the SNpr and putamen were evident. One interpretation for the lack of activation in the VTA or nucleus accumbens is that the current evoked pain fMRI paradigm does not engage mesocorticolimbic circuitry in a robust manner as it does nigrostriatal nodes (i.e., SNpr and putamen). Activation or sensitization of mesocorticolimbic circuitry hubs may be specific to a chronic pain state (Liu et al. 2025; Sunavsky et al. 2025; Baliki et al. 2010). Alternatively, the lack of significant VTA or nucleus accumbens BOLD responses during an acute pain state may stem from proper inhibition of these downstream RMTg targets (Bourdy and Barrot 2012). Moreover, we observed an increase in both RMTg-RNm and RMTg-LC correlations between 40°C and 46°C stimulation. While enhanced correlation of BOLD signals between the RMTg and LC during a pain state may stem from activation of the noradrenergic LC to RMTg pathway (Dornellas et al. 2021), causes for enhanced associations among the RMTg and RNm during evoked pain require further investigation.

Complementing these task-based observations, resting-state fMRI analysis of the human RMTg revealed a functional connectivity profile that closely resembles its known anatomical circuitry, with the strongest connections observed with the VTA, SN, red nucleus, and habenula. This functional connectivity is consistent with the role of the RMTg as a GABAergic gatekeeper of midbrain dopaminergic neurons and key node in the habenulo-dopaminergic aversive signalling pathway (Barrot et al. 2012). Significant coupling with the anterior cingulate cortex, insular cortex, and nucleus accumbens further anchors the human RMTg within mesolimbic circuitry involved in aversion, salience, and motivational control (Navratilova and Porreca 2014; Menon 2025). Beyond these targets, connectivity was observed with default mode network nodes including the medial prefrontal cortex, posterior cingulate cortex, and precuneus, suggesting a functional interface between brainstem dopaminergic systems and higher-order cortical networks involved in value representation and internally directed cognition (Menon 2023). Extensive RMTg-cerebellar connectivity was also observed and warrants further investigation in the human brain using reward-processing paradigms (Carta et al. 2019).

We note multiple limitations of this preliminary study. First, thermal stimulation fMRI studies, while performed at 7T, were performed in a small cohort of HCs. Relatedly, though 7T versus 3T fMRI provides enhanced spatial resolution and signal sensitivity, factors such as field inhomogeneities or head motion can still impact signal quality in the brain regions measured. To further understand the functional properties of the RMTg and its circuitry, future studies are not only required in a broader population of HCs but also in chronic pain conditions or neuropsychiatric conditions. Second, putative RMTg function was solely probed in the context of acute pain processing or an aversive state. Given the role of the RMTg in reward processing, encoding of negative reward prediction error, effort allocation, among other reward-related constructs, RMTg behaviour in human populations should be interrogated more broadly. Third, our study solely reports on evoked responses to 40°C and 46°C within the RMTg or resting-state RMTg functional connectivity. In follow-up studies, a characterization of effective connectivity centred around the RMTg would provide more concrete information on how the RMTg regulates its downstream mesocorticolimbic and nigrostriatal targets or how afferent signally from the HB or LC, for example, may alter RMTg function. Fourth, exploratory human postmortem analysis in a limited sample of donors showed diffuse expression of OPRM, NOP, and FOXP1 cells within an area adjacent to the decussation of the superior cerebellar peduncle, which contrasts with prior finding of dense GABAergic cell distribution in the putative RMTg (Filimontseva et al. 2026). This may indicate that delineation of GABAergic cell may more precisely identify the human RMTg. However, further molecular analysis of the human RMTg is warranted.

## Conclusion

This study provides preliminary evidence that an area within the human midbrain tegmentum, putatively identified as the RMTg, responds to noxious (i.e., 46°C) thermal stimulation as well as functional connectivity during the resting-state using 7T fMRI. The current results stemming from 7T fMRI data in humans translates findings on the RMTg from earlier rodent and non-human primate literature where the RMTg has been primarily characterized. Together, these translational findings support a model in which this region in human is implicated in nociceptive processing and integrated within previously defined reward-aversion circuitry in humans.

## Supplementary Material

Supplementary Files

This is a list of supplementary files associated with this preprint. Click to download.

RMTgSupplementaryMaterial063027.docx

## Figures and Tables

**Table 1:** Demographics, Quantitative Sensory Testing (QST), and Brief Pain Inventory.

Assessment	Measure	Mean	Standard Deviation
**Demographics**(12 males/10 females)	Age	32.59	9.79
**QST** Heat (°C)	Warm Detection	36.52	2.42
	Pain Detection	43.77	3.39
	Pain Tolerance	49.27	1.17
Cold (°C)	Cool Detection	29.60	1.58
**Verbal Pain Rating (fMRI)**	Pain Detection	11.67	9.67
*(0–10 pain scale)*	Pain Tolerance	3.27	6.55
**Brief Pain Inventory**	40°C	2.93	1.96
	46°C	6.02	2.04
	BPI – Worst	0.15	0.67
	BPI – Least	0.10	0.45
	BPI – Average	0.15	0.67
	BPI - Current	0.10	0.45

## Data Availability

Data available on request from the authors.
